# Potential implications of a therapeutic polyherbal infusion in Ayurveda as an adjunct to multidisciplinary relaxing techniques in alcohol withdrawal syndrome—an exploratory randomized study protocol

**DOI:** 10.3389/fmed.2026.1764758

**Published:** 2026-05-27

**Authors:** Adiveppal Nandini, Muraleedharan Devi, Esanamangalam Meera, Chandran Haritha, S. Sandeep, Leena Parameswaran Nair

**Affiliations:** 1 Department of Maulika Siddhanta (Basic Principles of Ayurveda), Amrita School of Ayurveda, Amrita Vishwa Vidyapeetham (University), Kollam, Kerala, India; 2 Department of Kayachikitsa (General Medicine), Amrita School of Ayurveda, Amrita Vishwa Vidyapeetham (University), Kollam, Kerala, India

**Keywords:** alcohol withdrawal, Ayurveda, CIWA-AR, counseling, herbal infusion, integrative medicine, *Kharjūrādi Mantha*, meditation

## Abstract

**Background:**

Alcohol withdrawal syndrome (AWS) constitutes a critical clinical outcome across the spectrum of alcohol use disorders (AUD), comprising severe neuropsychological manifestations during early abstinence among individuals with alcohol dependence. Pharmacological management primarily relies on benzodiazepines, which may cause dependence and hepatic stress. Addressing the core psychological domains is essential for uplifting the quality of life in such patients, and an integrated psychopharmacological approach rooted in holistic principles would be prioritized for better recovery. Ayurveda believes in treating the root cause rather than the symptoms, and hence, a polyherbal formulation is used in this trial, which would act on the doshas (humors) responsible for the manifestation of AWS. Hence, integrating the therapeutic intake of *Kharjuradi Mantha* coupled with mind–body relaxation interventions could provide substantial symptom control and enhanced recovery outcomes.

**Objectives:**

This exploratory randomized study protocol aims to evaluate the efficacy of a therapeutic herbal infusion (*Kharjuradi Mantha*) as an adjunct to multidisciplinary relaxation techniques comprising yoga, meditation, and counseling in individuals experiencing mild-to-moderate AWS.

**Methods:**

An exploratory, open-label, parallel-group randomized controlled trial will be conducted among 38 male inpatients who are diagnosed with mild-to-moderate AWS (Clinical Institute Withdrawal Assessment for Alcohol–Revised [CIWA-AR] score 8–15). Participants will be randomly assigned in a 1:1 ratio to receive either the herbal infusion in addition to relaxation techniques or the relaxation techniques alone for 28 days. The major outcome measure is to evaluate the difference in CIWA-AR scores from baseline to the 29th day after the completion of the trial in the trial setting.

**Expected outcomes:**

It is anticipated that the herbal infusion will enhance symptom resolution and overall psychological stability, which is reflected in the CIWA-AR scores when combined with yoga, meditation, and counseling, compared with standard non-pharmacological management alone.

**Clinical trial registration:**

https://ctri.nic.in, Identifier CTRI/2024/07/070952.

## 1 Introduction

Alcohol dependence remains a global health challenge, contributing to more than three million deaths annually and representing approximately 5% of the total global disease burden (1). Alcohol withdrawal syndrome (AWS) is a practically alarming ramification occurring due to intentional or unintentional sudden and abrupt cessation of binge drinking in patients suffering from alcohol use disorders (AUD) (2). Recent studies have indicated that up to 50% of patients with AUDs will experience AWS manifestations leading to substantial morbidity and mortality, along with approximately 5.1% of disability-adjusted life years (DALYs) (3). During early abstinence, individuals with AWS may present with minor-to-moderate systemic symptoms with the involvement of psychological domains such as headache, mild anxiety, insomnia, gastrointestinal discomfort, tremors, restlessness, sweating, and sleep disturbances. However, if untreated, these symptoms can progressively worsen into severe complications such as delirium tremens and withdrawal seizures, posing significant clinical risks4. Diagnosis and management can be challenging because of potential barriers in relation to accessibility and availability of services, as well as the public stigma, which discourages individuals to seek help or treatment.

The current management strategies primarily rely on benzodiazepines and supportive care to stabilize neurological and autonomic disturbances (4). Even though the protocol has been found to be effective in reducing acute symptoms, these pharmacological agents might often interact with ethanol, resulting in respiratory distress along with other effects such as sedation, dependence, and hepatic stress (4). Moreover, they do not adequately address the psychological domains of AWS, including craving, emotional instability, and relapse risk, which are vital in a sustained recovery.

Ayurveda is an indigenous, holistic, and culturally rooted medical system that has been widely practiced in the Indian subcontinent for centuries, with an extensive pharmacopeia for managing a wide range of ailments prevalent in the community. This system has emphasized the core concept of balance, wherein the cardinal etiology for the majority of the pathologies has been attributed to improper lifestyle regimens. Similarly, it has placed emphasis on an optimal amount of alcohol intake (Madyapaana), and one who indulges in irresponsible drinking would be contracted with loss of ojas (vital essence), leading to a condition called Madatyaya (alcoholic intoxication). Madya (Alcohol) possesses almost similar gunas (qualities) to visha (toxin) and may act similar to a toxin once it enters the body (5), thereby vitiating the dhatus (bodily tissues) and ojas present in the Hridaya (organ which is the root of rasavaha srotas and ojas, similar to heart). Abnormal levels of Madya may augment the intrinsic homologous qualities (guna) within the body, thereby impacting the equilibrium of vital humors or doshas. As Madya possesses ruksha (dry) and tikshna (sharp) qualities (6) it could essentially surge the normal levels of vata and pitta dosha (humors) as well. Clinically, these disproportionately higher levels of doshas should be counteracted with the opposite gunas. The resultant manifestations might continue even in the absence of alcohol, which would essentially require target-specific management prioritizing the Madya gunas and vitiated dosha-dhatus. Hence, a therapeutic polyherbal formulation, Kharjuradi Mantha, which can counter the vitiated doshas by imparting santarpana, i.e., a therapeutic nourishment to the vitiated body elements, has been selected in this study (7). Moreover, recent ethnopharmacological studies have demonstrated that *Phoenix dactylifera* (date fruit) and other nutritionally rich ingredients in Kharjuradi Mantha offer multiple restorative effects, including antioxidant and neuroprotective effects in alcohol-related oxidative stress and neuronal dysfunction (8). The spectrum of physical symptoms is intermingling with psychological features in all the stages, thereby manifesting emotional and behavioral decline in the patients of Madatyaya. Recent research evidence emphasizes that complementary interventions, particularly those involving mind–body relaxation techniques, have demonstrated promising results in improving emotional regulation, reducing craving, and promoting abstinence (9). Yoga, meditation, and counseling are widely recognized for their ability to modulate stress pathways, enhance parasympathetic activity, and support behavioral transformation during de-addiction therapy (10, 11). Moreover, the majority of rehabilitation centers in India follow the National Action Plan for Drug Demand Reduction (NAPDDR) Guidelines, which establish the fact that counseling and other relaxing techniques should be included under a standard protocol in such centers for mild-to-moderate AWS (12).

Despite the rising scopes in integrative approaches, there remains a significant lack of substantial clinical evidence evaluating the combined effect of therapeutic herbal remedies and structured relaxation techniques in the management of AWS. This phase will be more pronounced with uncomplicated physical symptoms and persistent appearance of psychiatric symptoms, which also need to be addressed as an area of greater concern. Hence, an evidence-based integrated protocol that targets both dimensions in an uncomplicated manner will help in reducing symptoms, thereby preventing their progression to a severe stage and uplifting the quality of life in the patients of AWS.

This exploratory randomized study protocol has been designed to inspect the efficacy and feasibility of an herbal infusion (Kharjuradi Mantha) administration when integrated alongside relaxation techniques such as yoga, meditation, and counseling, compared with relaxation techniques alone in the management of AWS. This trial aims to provide scientific evidence supporting an integrative, culturally compatible model for alcohol withdrawal management.

## 2 Methods and analysis

This study protocol has been drafted by adhering to SPIRIT (Standard Protocol Items: Recommendations for Interventional Trials) guidelines (13).

### 2.1 Study design

This is an open-label, parallel-group, exploratory randomized controlled trial designed to evaluate the efficacy and safety of a therapeutic polyherbal infusion (Kharjuradi Mantha) as an adjunct to multidisciplinary relaxation techniques in the management of mild-to-moderate AWS.

In the present study setting, multidisciplinary relaxation techniques constitute the baseline standard of care in the management of mild-to-moderate AWS in accordance with NAPDDR Guidelines. In moderate cases, these interventions will be continued under strict monitoring with a protocol for escalation to pharmacological management if required. A similar pre-defined pharmacological protocol with benzodiazepines is in place, and patients requiring such management will be excluded at baseline or managed accordingly by adopting the rescue medication strategy. This protocol does not aim to replace standard pharmacotherapy; however, it aims to evaluate the efficacy of the polyherbal infusion as an adjunct to the standard supportive care in patients with mild-to-moderate AWS.

### 2.2 Study setting

The trial will be conducted at the Spandana Integrated Rehabilitation Centre for Addicts (IRCA), Harihara, Karnataka, India.

### 2.3 Study population

According to the Diagnostic and Statistical Manual of Mental Disorders, Fifth Edition (DSM-5) criteria, participants who are male inpatients aged 25–60 years and diagnosed with mild-to-moderate AWS will be recruited for this trial11.

### 2.4 Eligibility criteria

The inclusion criteria for this trial include male participants aged between 25 and 60 years, those who are diagnosed with mild-to-moderate AWS (CIWA-AR score 8–15) (14), those who are willing to participate and provide written informed consent, and residents who are already undergoing non-pharmacological management for alcohol withdrawal. Patients with severe withdrawal (CIWA-AR > 15) or delirium tremens, hepatic failure, ischemic heart disease, or uncontrolled metabolic disorders; those who are currently on psychotropic or hepatotoxic medication; those who are diagnosed with any major psychiatric disorders; or those who are in concurrent participation with another clinical trial will be excluded from the study.

All participants will be strictly monitored and continuously evaluated for the progression of symptoms into a severe stage, where a predefined rescue medication protocol will be adopted for conventional management in such cases.

### 2.5 Recruitment

Participants will be recruited from the inpatient population of Spandana IRCA through clinician referrals and screening of eligible cases. Written informed consent will be obtained before enrolment. Patients will be added one at a time until the target sample size is achieved.

### 2.6 Interventions

**Figure 1:**
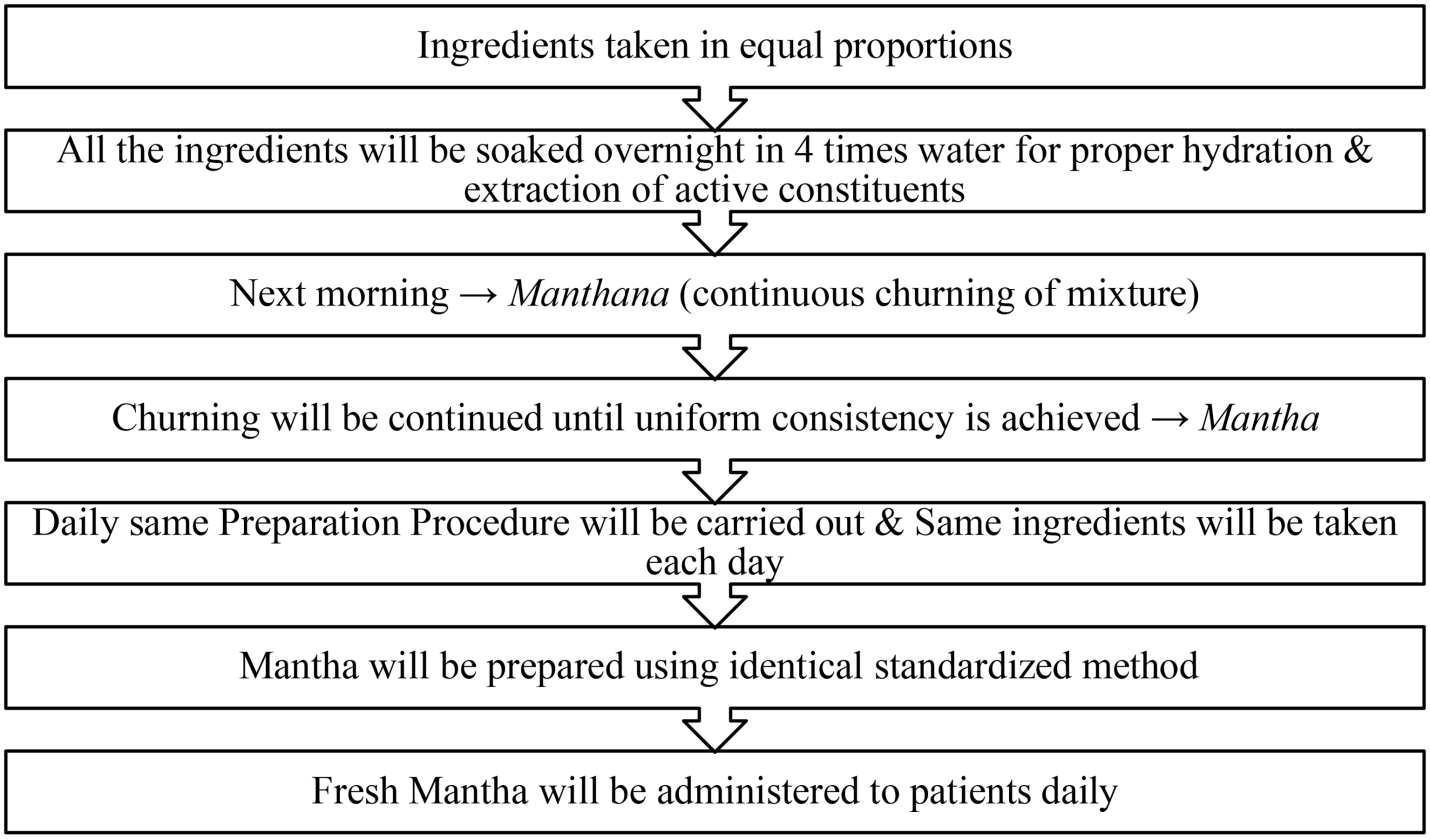
Method of preparation of *Kharjuradi Mantha.*

After admission, each patient will be given a 2–3-day adjustment period, depending on their mental status, to help them acclimatize to the environment. Following this, the inclusion and exclusion criteria will be assessed. The study details will be explained thoroughly, and only those patients who are willing to participate will be considered for enrolment. Before initiation of the trial, the CIWA-AR scale will be administered. Patients scoring less than 15, indicating mild-to-moderate alcohol withdrawal symptoms, will be confirmed as suitable candidates for the study. After obtaining their consent and explaining all trial procedures, these patients will be included in the trial. The ingredients will be botanically identified, and the infusion sample will be prepared from a CARE KERALAM GMP-certified company. The preparation will be replicated daily at the rehabilitation center under the direct supervision of a qualified quality pharmacy supervisor, strictly adhering to the master formula record and SOP established at CARE KERALAM. From day 1, participants in the trial group (Group A) will receive 50 mL of the Kharjuradi Mantha twice daily, administered 30 min before breakfast and dinner, along with 1 h of Yoga and meditation in the morning and 1 h of counseling daily. Ingredients mentioned for the polyherbal infusion Kharjuradi Mantha are mentioned in Table 1, and the general method of preparation and details of administration are demonstrated in Figure 1 and Table 2, respectively. The counseling sessions will be conducted by experts from the counseling department, while yoga and meditation sessions will be carried out by trained professionals. In the control group (Group B), patients will undergo yoga, meditation, and counseling only, without the formulation. The details of the intervention plan that will be followed in both groups have been provided in Tables 3, 4.

**Table 1:** Ingredients of polyherbal infusion *Kharjuradi Mantha.*

Sl. No.	Ingredients	Quantity
1	*Kharjura (Phoenix dactylifera)*	1 part
2	*Mrdveeka (Vitis vinifera)*	1 part
3	*Vrikshamla (Garcina indica)*	1 part
4	*Amlika (Tamarindus indica Linn)*	1 part
5	*Dadima (Punica granatum)*	1 part
6	*Parushaka (Grewia asiatica Auct)*	1 part
7	*Amalaki (Phyllanthus emblica)*	1 part
8	Water	4 parts

**Table 2:** Details of the administration of management.

Parameter	Details
Form	Freshly prepared *Kharjūrādi Mantha*
Dose	100 mL in two divided doses before breakfast and dinner (dose fixation according to classical reference)
Time of administration	Twice a day before food
Vehicle of administration	Water
Duration	For 4 weeks (28 days)

**Table 3:** Planned administration details of yoga and meditation in both groups.

Sl. No.	Type of practice	Practices		Duration
1	Prayer	Starting yoga with prayer		
2	Loosening practices	Fingers (clench fist and open), wrist (bending and rotation), elbow movements, shoulder rotation, neck movements (up-down, right and left movements), hip loosening exercise, knee flexion, ankle movement, toes movement		10 min
3	Breathing practices	*Anuloma viloma pranayama, ujjayi pranayama, bhramari pranayama*		10 min
4	*Yogasana* (simple yogic poses)	4.a	*Surya namaskara* (sun salutations)	10 min
		4.b	*Tadasana* (palm tree pose), *kateechakrasana* (standing spinal twist), *vrikshasana* (tree pose), *simhasana* (lion pose), *ardha matsyendrasana* (half spinal twist pose), *bhujangasana* (cobra pose), *shalabhasana* (locust pose), *dhanurasana* (bow pose), *pavanamuktasana* (wind- relieving pose), *vipareeta karani* (legs-up-the-wall pose) with *uddiyana bandha* (abdominal lock) and *moola bandha* (root lock), *shavasana* (corpse pose)	25 min
5	Meditation	“om” chanting		5 min
Total duration 60 min				

**Table 4:** Counseling session plan for both groups.

Type of counseling	Duration	Description
Individual counseling	1 h/session/day	One-to-one session with a counselor focusing on abstinence benefits, illness understanding, ill-health effects, social problems, motivation building, coping skills, and relapse prevention.
Family counseling	Weekly-1 h	Session with parents/partner explaining the importance of family support, communication improvement, identifying triggers at home, and helping the patient maintain abstinence.
Mass/group counseling	Weekly-1 h	Group session for sharing experiences, peer motivation, relapse prevention education, building confidence, and social support

**Figure 2:**
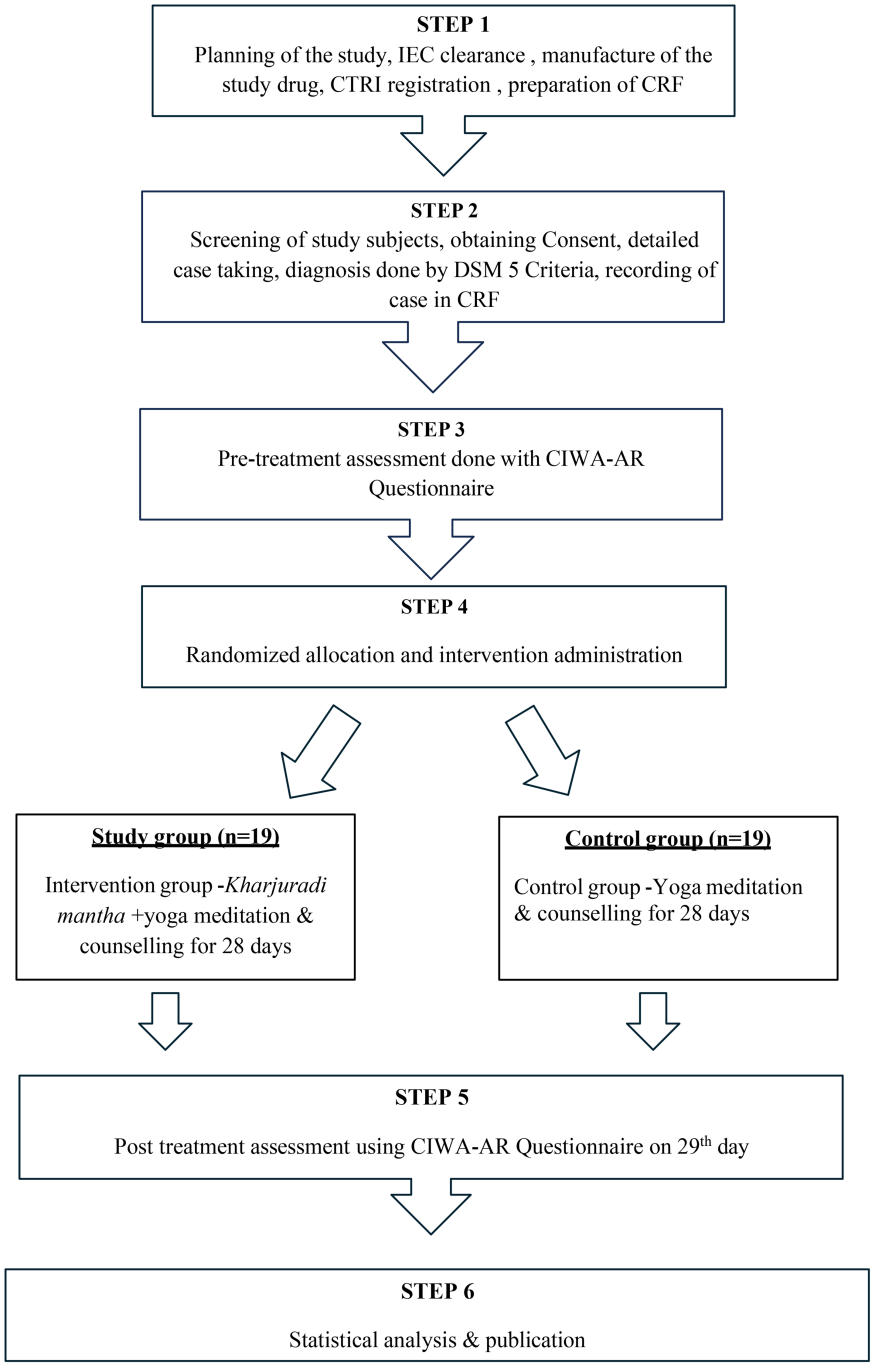
The trial methodology plan.

The trial methodology plan is given in Figure 2 and shows the detailed grouping, intervention, and assessments in the trial. This protocol will be followed for 28 days. On the 29th day, the CIWA-AR scale will be reassessed to evaluate post-intervention changes.

### 2.7 Outcome measures

The major outcome measure is to evaluate the changes in the CIWA-AR score from baseline to Day 28 to assess and diagnose the severity of alcohol withdrawal. The CIWA-AR scale has been a reliable and relevant tool in AWS, especially in clinical scenarios, where it has a sensitivity of 47% at a 95% confidence interval and a specificity of 88%, which makes it a reliable tool for assessment (15).

### 2.8 Participant timeline

Participants enrolled in the study will undergo three progress evaluations, as demonstrated in the planned schedule chart in Table 5. These evaluations encompass the gathering of preliminary information, administration of the intervention, and pre-post assessments. During the first evaluation (Day 0), screening and recruitment of patients will be conducted following diagnosis, along with assessment of eligibility criteria. Patients who do not meet the eligibility criteria or who are unwilling to participate will be excluded from the study. Eligible patients will be assessed using the CIWA-AR scale. On the same day, randomization will be performed using the lottery method, thereby allocating patients to either the study group or the control group, and the corresponding management will be initiated. During the second evaluation, progress will be reviewed after approximately 2 weeks, with specific attention to any adverse events in both groups. During the final evaluation (Day 29), both groups will be reassessed using the predetermined assessment criteria, and the findings will be documented for statistical analysis.

**Table 5:** Planned schedule chart for each participant in the trial.

Trial events	Day 0	2nd week	4th week (Day 29)
Screening & recruitment with informed consent	✓		
Inclusion & exclusion criteria assessment	✓		
Diagnostic criteria assessment	✓		
CRF filing/Physical examination	✓		
Randomization and allocation	✓		
CIWA-AR Assessment	✓		✓
Patient education	✓		
Review of adverse events		✓	✓

### 2.9 Sample size calculation

As it is an exploratory clinical trial, it exceeds common rules of thumb for these studies (≥ 12 per arm), and after considering non-probability/convenience sampling measures, the study sample size was determined as 38, with 19 participants each in the study group and control group for a trial period of 28 days.

### 2.10 Randomization and allocation concealment

Participants will be randomly assigned in a 1:1 ratio to either the study or control group using a simple randomization method (sealed chit system).

Allocation concealment will be maintained through opaque, sequentially numbered envelopes opened only after participant enrolment. The enrollment number of the participants will be printed on the top of the envelope, and a slip indicating the group will be kept inside the envelope. After the recruitment and collecting the baselines, the researcher will provide the envelope to the participants in the order of their enrollment number. After opening the envelope, participants will be allocated to the corresponding group as indicated on the slip.

### 2.11 Blinding

Due to the distinct appearance and taste of the herbal infusion, participant and investigator blinding is not feasible. However, data analysts and outcome assessors will remain blinded to group allocation for reducing bias.

### 2.12 Data collection and management

All the baseline data collected will be mentioned in the case file and in a case report form (CRF), which is specific for each participant, comprising history, examinations, and assessment scales. CIWA-AR is a 10-step questionnaire with well-documented reliability, reproducibility, validity, and feasibility in clinical settings and has been administered here to assess the symptoms. Trial adherence will be assured by scheduling subsequent visit reminders, and the follow-up data will be mentioned in the case file as well as the CRF. Even if a patient wants to withdraw from the trial or if they are not complying with the treatment, the case file and CRF will be documented for record purposes.

Data entry will be done as a soft copy in secured databases and recorded manually as a hard copy in the CRF. The values will be cross-checked by the co-investigator before uploading into the database, and all the data will be rechecked before submitting for final analysis.

### 2.13 Statistical analysis

Statistical analysis will be performed using IBM SPSS version 29.0 software. Data will be analyzed using appropriate statistical measures, including paired *t*-tests for within-group comparisons and independent *t*-tests for between-group comparisons, assuming normal distribution of data. Repeated-measures ANOVA will be applied to assess changes over time in multiple groups based on the nature of data, and outcome measures. A *p*-value of < 0.05 will be considered significant.

### 2.14 Safety monitoring and adverse events

Participants will be observed daily for adverse effects. All adverse events will be documented, assessed using WHO–UMC causality assessment, and managed appropriately (16).

### 2.15 Rescue medications

In case of rapid aggravation of symptoms, the trial will be stopped, and the participant will be referred to a tertiary health care center for advanced management, including pharmacological strategies.

## 3 Ethical considerations

This study protocol approval has been granted from the Institutional Ethics Committee of Tapovana Ayurvedic Medical College and Hospital, Doddabathi, Davanagere (Approval No. TAMC&H/IEC/01/2024–25, dated 16 July 2024). The trial is prospectively registered with the Clinical Trial Registry of India (CTRI/2024/07/070952).

The study will adhere to the Good Clinical Practice (GCP) guidelines according the Indian Council of Medical Research (ICMR), the Declaration of Helsinki (2013 revision), and the National Ethical Guidelines for Biomedical and Health Research on Human Participants (2017) by ICMR. Patient information leaflets with a comprehensive outlining of the objectives and outcomes of the research will be provided in two languages (English/Kannada). Voluntary written informed consent will be obtained from all participants. Confidentiality of the participant information will be maintained throughout the trial, and data will be securely stored in password-protected databases. Patient enrollment number will be used as part of the identification of the patient during data upload and analysis. Only the Principal Investigator and co-investigator will have access to research data. Safety of the intervention will be prioritized by monitoring and recording any of the adverse events that occur during the trial, followed by notification to the Institute Ethical Committee (IEC). Findings will be published through peer-reviewed journals, conferences, and institutional reports by considering all the scientific, ethical, and safety aspects of the trial.

### 3.1 Ancillary and post-trial care

There have been no ancillary studies proposed in the present clinical study. Participants will continue to be provided with the routine standard of care, which is followed in the study setting.

## 4 Discussion

This exploratory randomized study aims to evaluate the efficacy of a therapeutic herbal infusion (Kharjuradi Mantha) as an adjunct to yoga, meditation, and counseling in the management of AWS. With rising incidents of clinical challenges and escalation into severe complications, there is an increasing interest in alternative approaches, which include the integration of herbal remedial measures with multidisciplinary relaxation techniques in the uncomplicated stages of AWS. Establishing a pharmacological basis backed with safe and effective findings may contribute to the growing literature that supports an integrative management framework in this condition.

Ayurveda firmly establishes the principle of “countering with the opposite” where the properties of ingredients in a medicine or preparation could act against the doshas (morbid factors) of a disease (17). A majority of the ingredients of the polyherbal infusion “Kharjuradi Mantha” comprise Guru (heavy), Sheeta (cold), and Snigdha (unctuous) Gunas (qualities), which could combat the impact of Laghu (light) Ushna (hot), and Tikshna (sharp) gunas of Madya (alcohol) in AWS. Hence, this would enhance the metabolism and nourishment through Agnidipana and Tarpana gunas of the ingredients, along with the regulation of the movement of Vata as well as Pitta dosha. The majority of the ingredients of Kharjuradi Mantha have vatapitta shamaka properties and are predominant with madhura-amla rasa, which is beneficial in combatting the properties of Madya (18, 19). Other concomitant relaxation techniques would be concentrating on the satva and optimizing the manodoshas (Rajas and Tamas), thereby encompassing the psychological aspects of Madatyaya (20).

Alcohol induces long-term detrimental changes in neurons after repeated detoxification (which is the “kindling phenomenon”), along with brain hyperexcitability caused by abrupt alcohol cessation that upregulates the NMDA receptors (21). Individual pharmacological activities exerted by the bioactive principles of each ingredient collectively constitute the reduction in the symptoms of AWS as well as alcohol-induced systemic toxicities. Various phytopharmacological reviews of the main ingredient Kharjura (*Phoenix dactylifera*) demonstrate anti-inflammatory and antioxidant activity by free radical scavenging action and hepatoprotective activity by effectively reducing the hepatic markers, thereby contributing to potential regulation in AWS (22). Moreover, all the other ingredients also possess the same pharmacological activities, whereas Dadima (Punica grantum) and Amalaki (*Emblica officinalis*) possess an additional neuroprotective action, which could rectify the pathophysiological framework of AWS (23, 24). Several preclinical studies suggest that resveratrol, which is a common constituent in Draksha (*Vitis vinifera*), enhances GABAergic inhibition, possesses anxiolytic activities, and downregulates glutamate-induced cytotoxicity, which leads to hyperexcitation in AWS (25). The major ingredient Kharjura (*Phoenix dactylifera*) and polyphenols in other ingredients like Dadima ameliorate glutamate-induced excitotoxicity, which could be highly contributing to the rectification (26).

Umhau et al. studied the cerebrospinal fluid levels of monocyte chemoattractant protein-1 (MCP-1), as well as interleukin-1β along with tumor necrosis factor-*α* in 28 alcoholics and 13 healthy volunteers, and reported higher levels of MCP-1 in alcoholics, which were positively associated with peripheral markers of alcohol-induced liver inflammation, i.e., serum gamma-glutamyl transferase and aspartate aminotransferase levels in alcoholics. This finding necessarily indicates the elevated inflammatory marker, MCP-1, in accordance with the neurodegeneration and neuroinflammation induced by alcohol (27). Analytical studies of all the ingredients have suggested anti-inflammatory activities of the phytoconstituents in almost all ingredients, which would potentially make it an ideal therapeutic alternative in the management of AWS (23, 28–30).

Concurrently, yoga and meditation promote autonomic balance and psychological stability, while counseling supports behavioral modification30. Moreover, relaxation techniques such as yoga have been found to improve the body’s ability to manage stress, combat inflammatory cytokines, and promote a sense of well-being, which could combat the effects of kindling in AWS (31, 32). Hence, this integrated approach seeks to enhance recovery outcomes, reduce withdrawal severity, and provide a non-dependence-forming alternative to conventional pharmacotherapy. This trial focuses on exploring the efficacy of an augmented approach when a polyherbal infusion of multifaceted action is coupled with the pre-established potency of various relaxing techniques for AWS.

Nevertheless, this trial design has some constraints with respect to its applicability in a larger population. Being an exploratory randomized trial, our primary aim is to generate preliminary-level evidence for further larger trials with multiple arms in a much larger population. The sample size of the study is 38, which would not meet the required criteria for external validity. As it is a time-bound and exploratory study design, non-probability sampling measures were employed, and hence, a small sample size was adopted in this study. Moreover, as there is an academic time limit, assessing the long-term sustained efficacy would not be possible, which could only be carried out by a large sample-sized clinical trial in a prospective cohort setup. Participant and investigator blinding is not feasible, as the appearance and taste could vary while preparing the trial infusion. This study is essentially concentrated on the physical and psychological withdrawal effects in mild and moderate AWS rather than alcohol-related multiple systemic effects, including hepatoprotective effects. Including the assessment of these effects would require much more sophisticated methodologies, and it is beyond the scope of this study.

## 5 Conclusion

The outcomes of this trial are expected to deliver sound background clinical evidence regarding the therapeutic efficacy of a polyherbal infusion integrated with multidisciplinary relaxation techniques in improving the subjective presentations and signs in participants with AWS. Anticipated findings could be used as a reference tool for providing a scientific platform for more sophisticated trials incorporating neurochemical and psychometric parameters and thereby exploring the scope of Ayurveda in implementing therapeutic protocols and evidence-based rehabilitation of AWS.
